# Complete mitochondrial genome sequence data of *Pterygoplichthys gibbiceps* (Actinopterygii, Loricariidae)

**DOI:** 10.1016/j.dib.2026.112963

**Published:** 2026-06-11

**Authors:** Thu Thao Thi Huynh, Phi Anh Ngoc Nguyen, Nga Thi Nguyen, Minh Trong Quang

**Affiliations:** a Department of Hematology, Faculty of Medical Laboratory, Hong Bang International University, Ho Chi Minh City 70000, Vietnam; b School of Medicine, Tan Tao University, Tay Ninh 82700, Vietnam; c Department of Microbiology - Parasitology, School of Pharmacy, University of Medicine and Pharmacy at Ho Chi Minh City, Ho Chi Minh City 70000, Vietnam

**Keywords:** Invasive species, Leopard pleco, Loricariid catfish, Mitogenome, Molecular phylogenetics

## Abstract

*Pterygoplichthys gibbiceps* (Kner, 1854) is a loricariid catfish (Actinopterygii: Loricariidae) recognized as an invasive alien species introduced through the ornamental fish trade. The species has established invasive populations and has been recorded from multiple localities across Vietnam. Here, we presented the complete mitochondrial genome data of *P. gibbiceps* from Vietnam, generated by high-throughput sequencing. The mitogenome contains 13 protein-coding genes, 22 transfer RNA genes, two ribosomal RNA genes, and a control region. Comparative analyses revealed a high degree of conservation among *Pterygoplichthys* mitogenomes in genome structure, gene content, gene order, and codon usage patterns. In addition, all mitochondrial protein-coding genes of *P. gibbiceps* exhibited Ka/Ks values below 1, indicating predominant purifying selection relative to other congeners. Phylogenetic reconstruction supported the monophyly of *Pterygoplichthys* and placed *P. gibbiceps* as an early-diverging lineage within the genus. These mitochondrial genome data provide a valuable resource for future studies on species identification, population structure, and evolutionary relationships of *P. gibbiceps* in Vietnam and elsewhere, and also contribute to comparative mitochondrial genomic studies in armored catfishes.

Specifications Table

**Table:** 

Subject	Biology
Specific subject area	Omics: Genomics.
Type of data	Table, Figures. Raw, Analyzed.
Data collection	Total genomic DNA of *Pterygoplichthys gibbiceps* was extracted using a DNeasy Blood & Tissue Kit. The NEBNext Ultra II DNA Library Prep Kit for Illumina was used for library preparation, followed by high-throughput sequencing on the MGI BISEQ-99 platform using 150 bp paired-end reads. The resulting sequence data were assembled *de novo* using NOVOPlasty v4.3.5, and mitochondrial genome annotation was performed using the MITOS2 tool available via the Galaxy web server. The complete circular mitogenome of *P. gibbiceps* was visualized using the OGDRAW tool.
Data source location	Location: Van Hoa Lake, Son Hoa, Dak Lak Province. Country: Vietnam. Latitude and longitude: 13°10′01.8"N 109°07′48.7"E The voucher specimen was deposited at the Department of Microbiology - Parasitology, School of Pharmacy, University of Medicine and Pharmacy at Ho Chi Minh City (contact: Minh Trong Quang, qtminh@ump.edu.vn) under voucher number UMP_2025_10_VN.
Data accessibility	Repository name: GenBank, NCBI Data identification number: PX583372 Direct URL to data: www.ncbi.nlm.nih.gov/nuccore/PX583372
	The associated BioProject, SRA, and BioSample numbers are PRJNA1368433, SRR36145364, and SAMN53364641, respectively.
Related research article	None

## 1 Value of the Data

• This study provides a complete, annotated mitochondrial genome of *P. gibbiceps*, a loricariid catfish widely distributed in the ornamental fish trade, serving as a standardized genomic reference at the gene, codon, and genome levels.
• The dataset enables researchers to reconstruct phylogenetic relationships within Loricariidae and other Siluriformes by concatenating the provided protein-coding gene sequences with existing mitogenomic resources.
• Additionally, our mitogenomic data can support the development of species-specific primers and probes for DNA barcoding, metabarcoding, environmental DNA detection, and population-level surveys of both native and non-native habitats.

## 2 Background

The genus *Pterygoplichthys* comprises South American armored catfishes native to the Amazon and Orinoco basins. Owing to the aquarium trade, species of this genus have been widely introduced worldwide and are now established as invasive taxa on five continents. These algae-eating fish are morphologically similar, which can complicate species-level identification, and they can negatively impact native ecosystems by destabilizing banks and competing with indigenous fauna [1]. In Vietnam, *Pterygoplichthys* species have been reported from freshwater habitats across the country, including southern, central, and, to a lesser extent, northern regions. Invasive populations were first documented in southern Vietnam, particularly in the Dong Nai River, in 2000 [2], and subsequently in central Vietnam (Khanh Hoa Province) [3] and the Red River in northern Vietnam [4], indicating a broad geographic expansion. The invasive capacity of this species is associated with its high environmental tolerance, broad diet, and strong reproductive capacity, all of which may increase ecological pressure on native freshwater communities in Vietnam [[2], [3], [4]].

*Pterygoplichthys gibbiceps*, also known as the sailfin or leopard pleco, naturally inhabits the Orinoco and Amazon River basins of northern South America [5]. Within its native range, the species is of considerable ornamental value, and wild-caught individuals are exported through the aquarium trade, contributing to local economies. Like other members of the genus, *P. gibbiceps* has been introduced beyond its native range and was reported as the first loricariid fish recorded in Poland [6]. Despite its broad distribution and ecological relevance, genomic resources for *P. gibbiceps* remain limited, and until now, no complete mitochondrial genome has been reported for the species. Mitogenomes of related taxa, such as the 16,425 bp mitogenome of *P. pardalis*, have been characterized in recent studies, supporting the phylogenetic placement of *Pterygoplichthys* within Loricariidae [7]. The present study fills a critical gap in Loricariidae genomic data and provides a valuable molecular resource for species identification, phylogenetic and taxonomic research, and evolutionary and invasive biology studies of this ecologically significant species.

## 3 Data Description

**Fig. 1:**
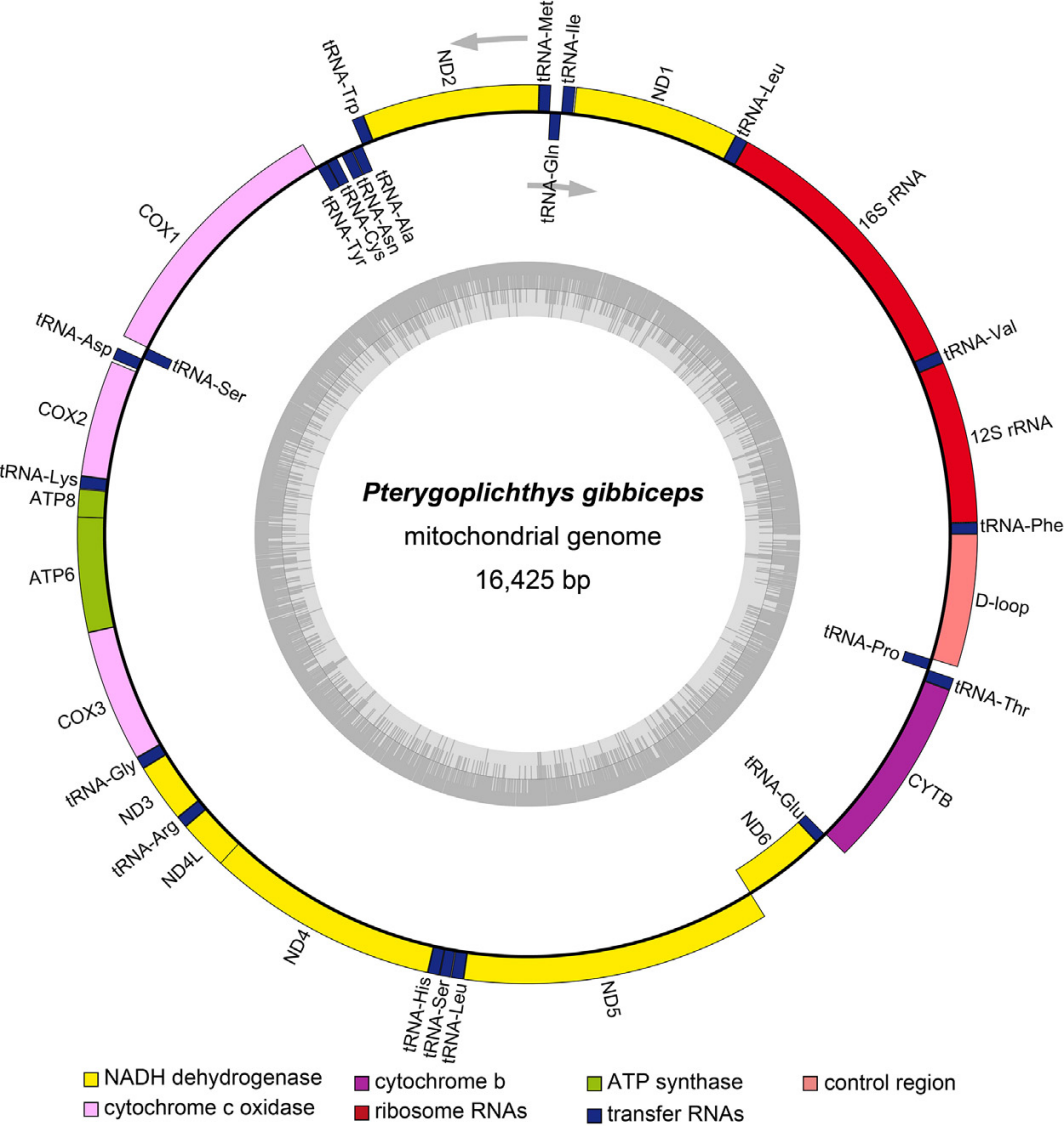
Circular mitogenome map of *Pterygoplichthys gibbiceps* generated in this dataset.

**Fig. 2:**
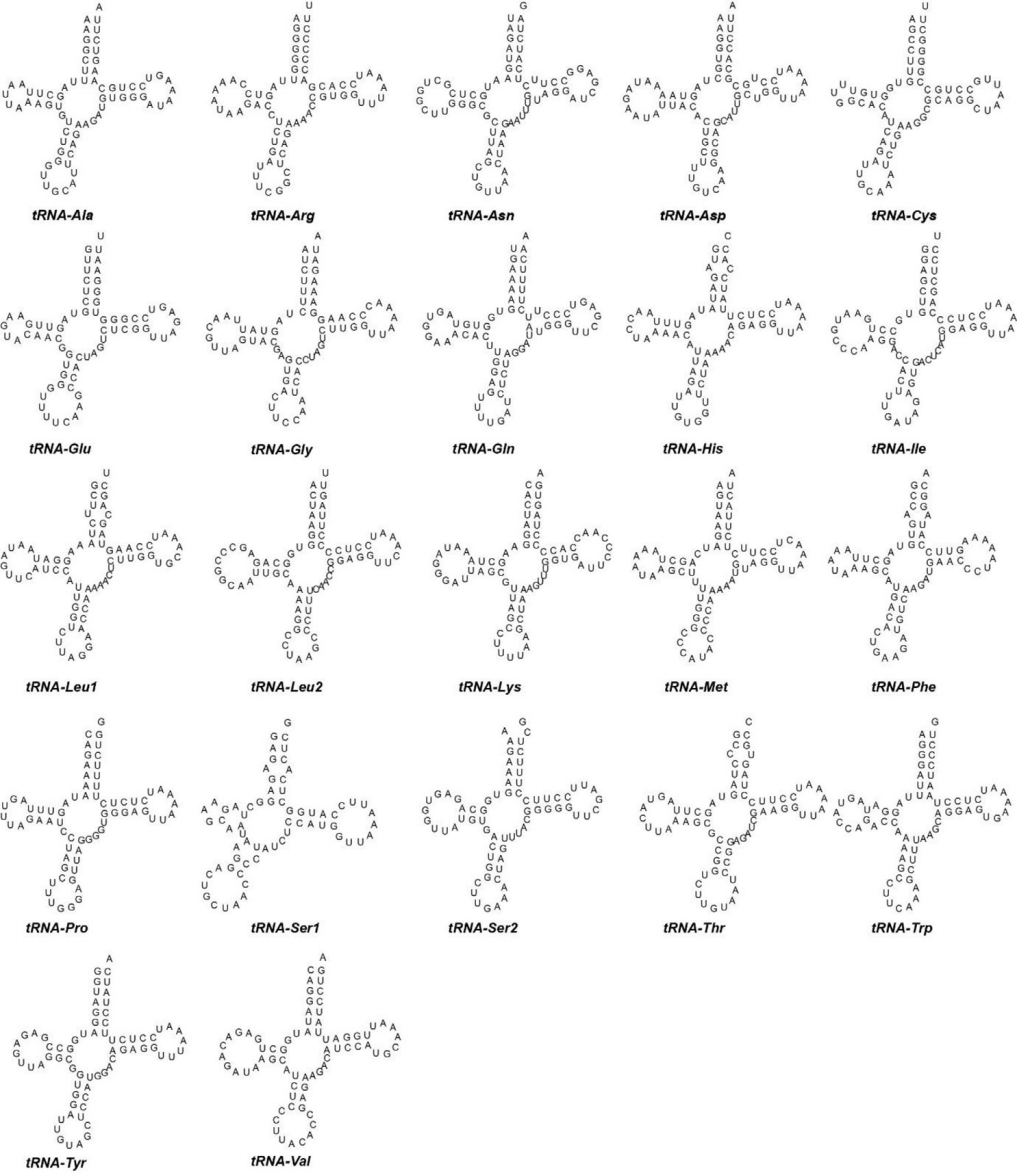
Predicted secondary structures of the 22 mitochondrial tRNA genes in the mitogenome of *Pterygoplichthys gibbiceps*.

The *P. gibbiceps* mitochondrial genome was a circular DNA molecule of 16,425 bp (Figs. 1; S1). The overall GC content was 41.6%, while the AT content is 58.4%, with a nucleotide composition of A: 31.5%, T: 26.9%, C: 26.8%, and G: 14.8%. The genome encoded 37 genes, including 13 protein-coding genes (PCGs), 22 transfer RNA (tRNA) genes, two ribosomal RNA (rRNA) genes, and a control region (Fig. 2). Among these, nine genes, including one PCG (*ND6*) and eight tRNAs (*tRNA-Gln, tRNA-Ala, tRNA-Asn, tRNA-Cys, tRNA-Tyr, tRNA-Ser, tRNA-Glu*, and *tRNA-Pro*), were encoded on the complementary strand (Table 1).

**Table 1:** Annotation of the mitochondrial genome of *Pterygoplichthys gibbiceps.*

Genes	Strand	Position	Length (bp)	Intergenic nucleotide (bp)	Start codon	Stop codon
*tRNA-Phe*	+	1-68	68	0		
*12S rRNA*	+	69-1022	954	0		
*tRNA-Val*	+	1023-1094	72	0		
*16S rRNA*	+	1095-2770	1676	0		
*tRNA-Leu*	+	2771-2845	75	0		
*ND1*	+	2846-3820	975	0	ATG	TAA
*tRNA-Ile*	+	3825-3896	72	4		
*tRNA-Gln*	-	3897-3967	71	0		
*tRNA-Met*	+	3967-4036	70	-1		
*ND2*	+	4037-5081	1045	0	ATG	T–
*tRNA-Trp*	+	5082-5152	71	0		
*tRNA-Ala*	-	5155-5223	69	2		
*tRNA-Asn*	-	5225-5297	73	1		
*tRNA-Cys*	-	5331-5397	67	33		
*tRNA-Tyr*	-	5398-5467	70	0		
*COX1*	+	5469-7019	1551	1	GTG	TAA
*tRNA-Ser*	-	7020-7090	71	0		
*tRNA-Asp*	+	7095-7167	73	4		
*COX2*	+	7182-7872	691	14	ATG	T–
*tRNA-Lys*	+	7873-7946	74	0		
*ATP8*	+	7948-8115	168	1	ATG	TAA
*ATP6*	+	8106-8789	684	0	ATG	TA-
*COX3*	+	8789-9572	784	0	ATG	T–
*tRNA-Gly*	+	9573-9644	72	0		
*ND3*	+	9645-9993	349	0	ATG	T–
*tRNA-Arg*	+	9994-10063	70	0		
*ND4L*	+	10064-10360	297	0	ATG	TAA
*ND4*	+	10354-11734	1381	-7	ATG	T–
*tRNA-His*	+	11735-11804	70	0		
*tRNA-Ser*	+	11805-11871	67	0		
*tRNA-Leu*	+	11873-11945	73	1		
*ND5*	+	11946-13772	1827	0	ATG	TAA
*ND6*	-	13769-14290	522	0	ATG	TAG
*tRNA-Glu*	-	14291-14359	69	0		
*CYTB*	+	14362-15499	1138	2	ATG	T–
*tRNA-Thr*	+	15500-15572	73	0		
*tRNA-Pro*	-	15571-15640	70	-2		
D-loop	+	15641-16425	785	0		

Note: (+) and (−) indicate genes encoded on the forward and reverse strands, respectively. Positive values in the intergenic nucleotide column indicate spacer regions, whereas negative values indicate overlaps between adjacent genes. Incomplete stop codons (T– or TA-) are presumed to be completed by post-transcriptional polyadenylation.

All PCGs begin with the standard ATG start codon, except for *COX1*, which initiated with the GTG. Seven PCGs (*ATP6, COX2, COX3, CYTB, ND2, ND3,* and *ND4*) terminated with an incomplete stop codon T, a common feature in vertebrate mitogenomes. The mitogenome also contained 10 intergenic spacer regions, ranging from 1 to 33 bp, with the longest located between *tRNA-Asn* and *tRNA-Cys*. Additionally, three overlapping regions were identified, ranging from 1 to 7 bp in length, with the longest overlap occurring between *ND4L* and *ND4* (Table 1).

A total of 22 typical mitochondrial tRNA genes were identified in the *P. gibbiceps* mitogenome. Their lengths ranged from 67 to 75 bp, with *tRNA-Cys* and *tRNA-Ser* being the shortest (67 bp), and *tRNA-Leu* the longest (75 bp). The genome also contained two copies of each of *tRNA-Leu* and *tRNA-Ser*, as is commonly found in vertebrate mitochondrial genomes. The secondary structures of the 22 mitochondrial tRNAs of *P. gibbiceps* were predicted and are shown in Fig. 2. Most tRNAs exhibited the typical cloverleaf structure. At the same time, *tRNA*-*Ser1* displayed a reduced DHU arm, a feature commonly reported in vertebrate mitogenomes.

**Fig. 3:**
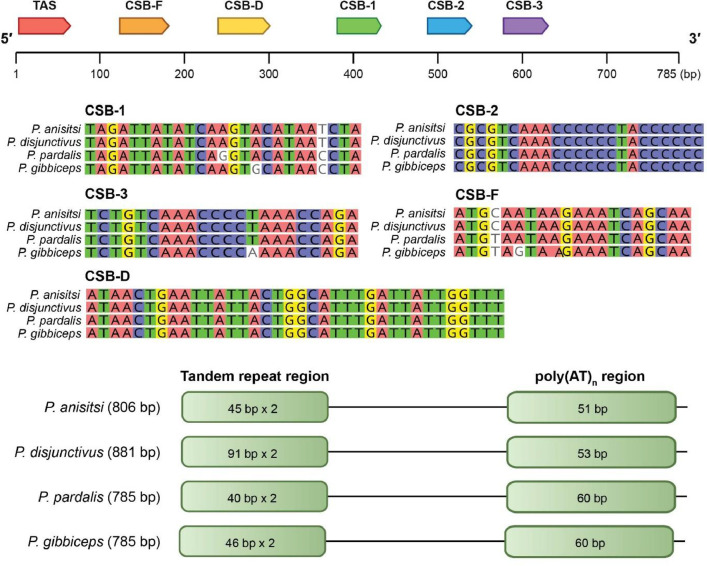
Mitochondrial control region structure and conserved motifs in *Pterygoplichthys* species. Conserved sequence blocks (CSB-F, CSB-D, CSB-1, CSB-2, and CSB-3) are indicated along the 785-bp control region of *Pterygoplichthys gibbiceps* mitogenome. The middle panels show multiple sequence alignments of CSB-1, CSB-2, CSB-3, CSB-F, and CSB-D among *P. anisitsi, P. disjunctivus, P. pardalis*, and *P. gibbiceps*. The lower panel summarizes species-specific tandem repeat regions and poly(AT)_n_ regions within the control region. Repeat unit lengths and copy numbers are shown for each species, with *Pterygoplichthys* species containing a duplicated tandem repeat and a poly(AT)_n_-rich region.

The mitochondrial control region of *P. gibbiceps* was 785 bp in length and was located between the flanking tRNA-Pro and tRNA-Phe genes (Fig. 3). Its predicted secondary structure is shown in Fig. S2. Annotation of the control region revealed a TAS-associated domain at the 5′ end, conserved sequence blocks (CSBs) in the central region, and an AT-rich repetitive tract toward the 3′ end. In detail, the TAS-associated domain was identified at positions 1–82, followed by CSB-F at positions 140–159, CSB-D at positions 219–254, CSB-1 at positions 452–476, CSB-2 at positions 525–559, and a CSB-3-like motif at positions 569–590 in the *P. gibbiceps* mitogenome. Comparative alignment with other *Pterygoplichthys* species showed that these CSB motifs were conserved, although minor changes were observed in CSB-2 and CSB-3 across species. In addition, *P. gibbiceps* mitogenome contained a tandem repeat region consisting of 46-bp repeat units and a 60-bp poly(AT)n-rich region. These structural features indicate that the control region of *P. gibbiceps* retains conserved regulatory motifs and harbors repeat-rich segments that may contribute to sequence length variation among closely related species.

**Fig. 4:**
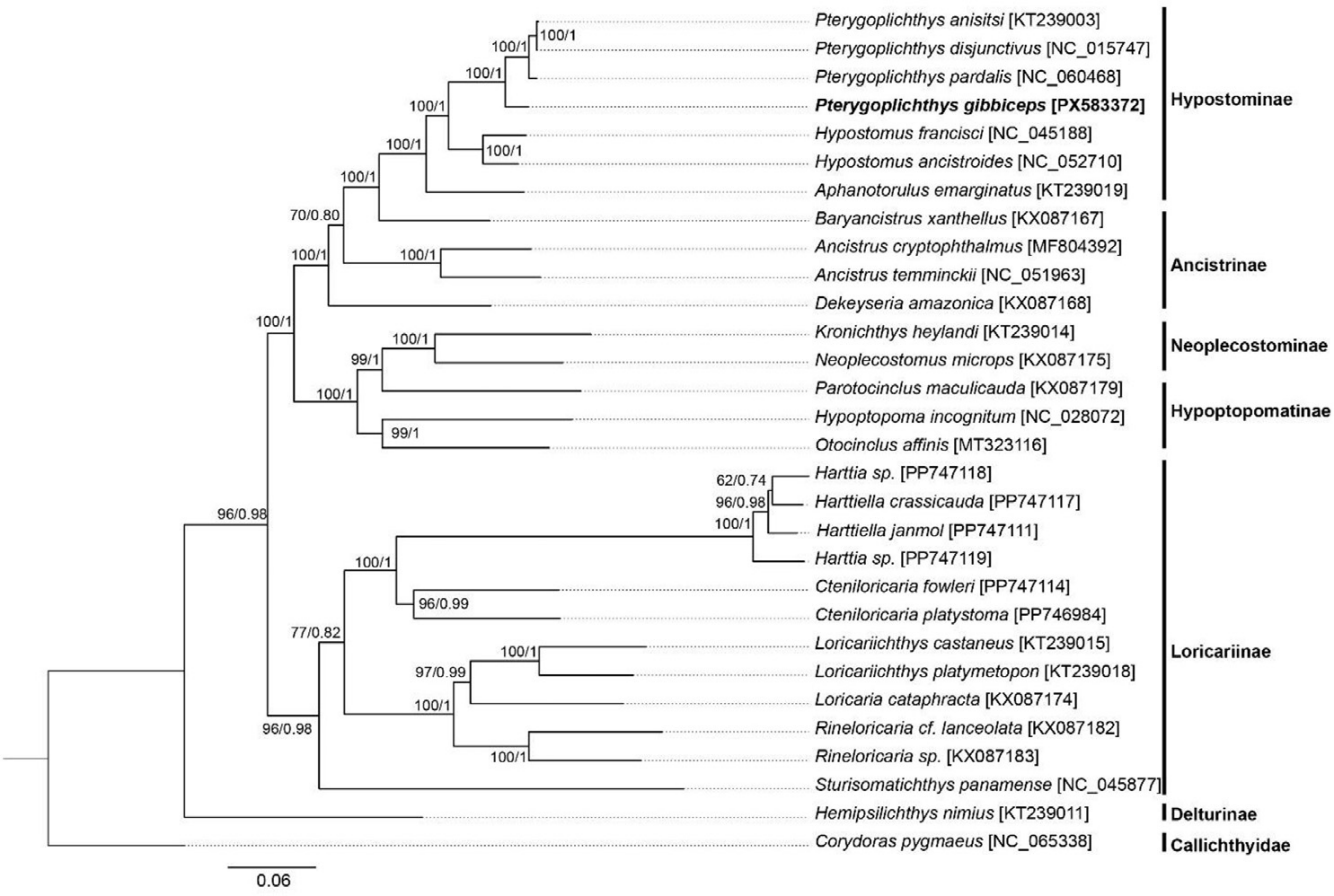
Phylogenetic relationships of *Pterygoplichthys gibbiceps* within Loricariidae inferred from whole mitochondrial genome data. Support values shown at nodes are maximum-likelihood bootstrap percentages/Bayesian posterior probabilities. Accession numbers are provided in brackets after each taxon name, and the newly sequenced *P. gibbiceps* mitogenome is highlighted in bold. Names of loricariid subfamilies are indicated on the right.

Our phylogenetic analyses based on complete mitochondrial genomes recovered a well-resolved, strongly supported topology for the family Loricariidae, with most nodes receiving high support (bootstrap: 96–100 and posterior probability: 0.98–1.00). Both maximum-likelihood (ML) and Bayesian inference (BI) trees consistently showed that *Pterygoplichthys* species formed a well-supported monophyletic clade (Fig. 4). Within this clade, *P. gibbiceps* was placed as one of the earliest-diverging lineages (bootstrap: 100 and posterior probability: 1.00), suggesting a basal evolutionary position relative to its congeners.

## 4 Discussion

*P. gibbiceps* is a sailfin, suckermouth armored catfish with heavy bony plates and a very tall dorsal fin (>9 rays, often with a predorsal hump) [8]. It is protected by robust, stridulating pectoral-fin spines and is mainly regarded as a puncture/injury hazard [9]. The species has been introduced largely through aquarium releases and, together with other *Pterygoplichthys* species, has established in many non-native regions, where burrowing can increase turbidity, accelerate bank erosion, and disrupt benthic communities [9,10]. Although a recent taxonomic synthesis recognizes 15 valid *Pterygoplichthys* species [1], only three complete mitogenomes were previously available in public databases. Here, we presented the first complete mitochondrial genome of *P. gibbiceps*. The circular genome was 16,425 bp and contained the typical teleost gene set (13 protein-coding genes, 22 tRNAs, two rRNAs, and a control region), consistent with the conserved ∼16–17 kb organization of fish mitogenomes. Outside the control region, intergenic spacers were generally minimal (0–4 bp), except for a 14 bp interval between *tRNA-Asp* and *COX2* and a 33 bp interval within the WANCY tRNA cluster between *tRNA-Asn* and *tRNA-Cys*. Furthermore, compactness is maintained by several overlaps, including *ND4L*/*ND4* (7 bp), as well as multiple short (1–2 bp) overlaps common in fish mitochondrial genomes [11,12].

**Fig. 5:**
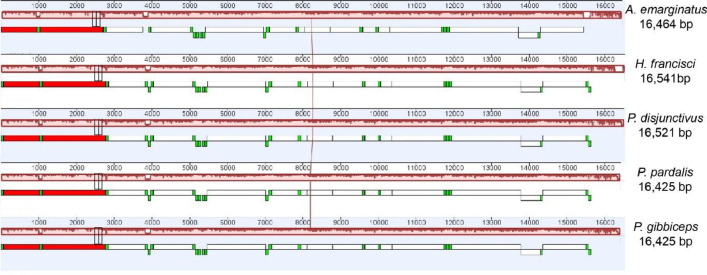
Comparative visualization of complete mitochondrial genomes among five loricariid catfishes, including *Aphanotorulus emarginatus, Hypostomus francisci, Pterygoplichthys disjunctivus, Pterygoplichthys pardalis*, and *Pterygoplichthys gibbiceps*. Genomes were linearized and aligned, and nucleotide similarity is shown by the red identity plot along genome coordinates (bp). Gene features are indicated below the plot, with rRNA genes in red, tRNA genes in green, and protein-coding regions in white. The names and lengths of the mitogenomes for each species are shown on the right.

Comparative mitogenomic analysis showed that *P. gibbiceps* had a largely conserved mitochondrial genome organization relative to the loricariids examined, with only slight length variation among taxa (16,425–16,541 bp) (Fig. 5). The identical genome length of *P. gibbiceps* and *P. pardalis* (16,425 bp) was consistent with published mitogenomes of *P. pardalis, Ancistrus emarginatus*, and *Hypostomus francisci*, which retained the standard vertebrate mitochondrial gene complement and a similar genome size [12,13]. Whole-genome alignments further indicated that the *P. gibbiceps* mitogenome was highly conserved relative to congeners, with differences mainly limited to scattered substitutions and a few short indel-rich regions, and the strongest divergence occurred in the control region. Overall, these results highlighted a common mitogenomic pattern in armored catfishes: conserved gene order and coding sequences, with most variation concentrated in the control region.

**Fig. 6:**
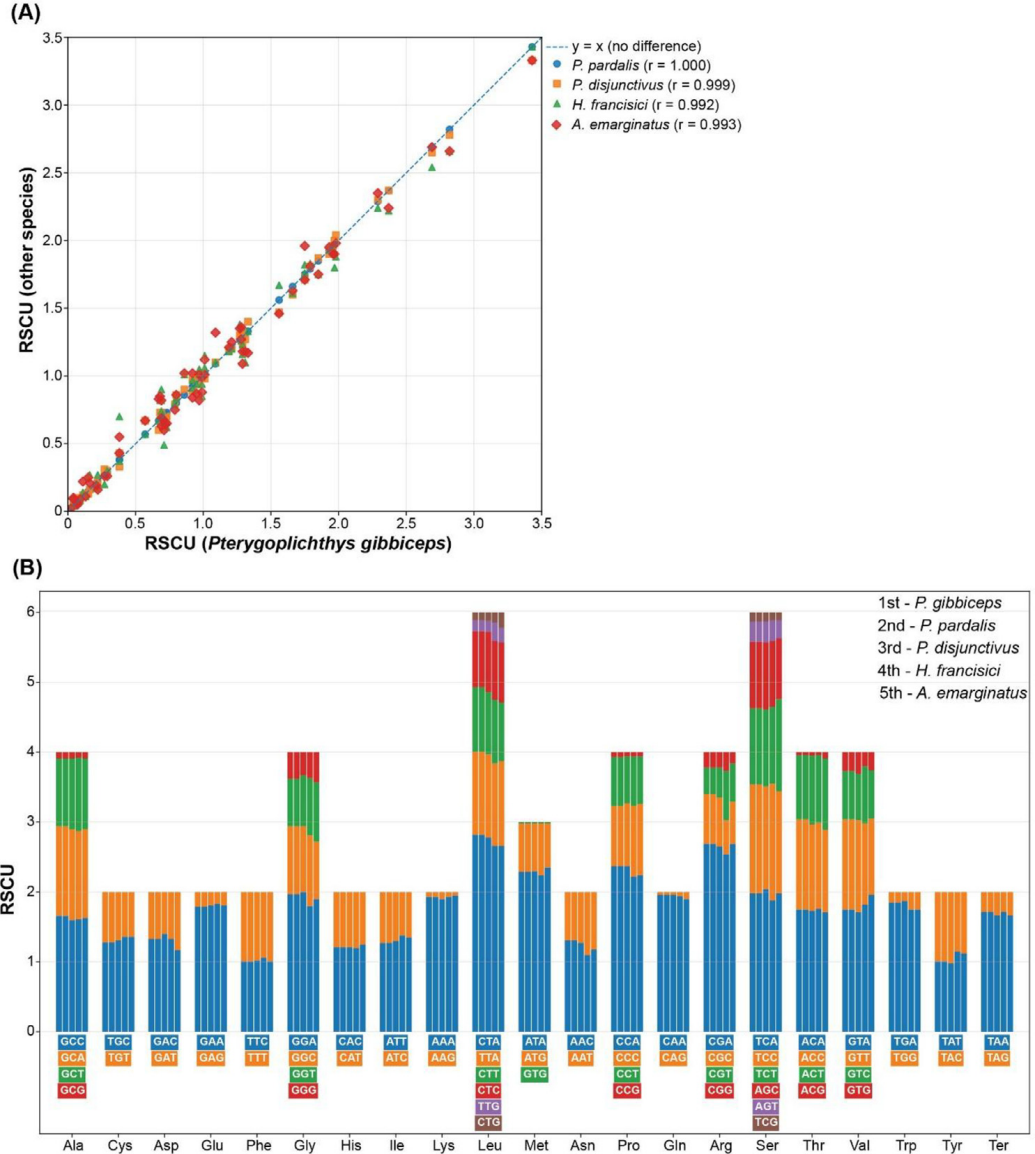
Codon usage comparison among five Loricariidae species based on RSCU values. (A) Scatter plots comparing codon-level RSCU values of *Pterygoplichthys gibbiceps* with each of the other four species: *Pterygoplichthys pardalis, Pterygoplichthys disjunctivus, Hypostomus francisci*, and *Aphanotorulus emarginatus*. Each point represents one codon; the dashed line indicates *y = x* (no difference). Pearson correlation coefficients (r) are shown in the legend. (B) Stacked bar chart of RSCU values grouped by amino acid. Within each amino-acid group, columns are ordered (left to right) as: 1^st^, *P. gibbiceps*; 2^nd^, *P. pardalis*; 3rd, *P. disjunctivus*; 4th, *H. francisci*; 5th, *A. emarginatus*. Colored segments correspond to synonymous codons (codon labels shown below each group).

Across the 64 codons, *P. gibbiceps* showed an overall codon composition similar to the four other loricariids (Fig. 6). We counted 3,802 codons in *P. gibbiceps* (including stop codons), which matched the totals in the other species (3,801–3,802). RSCU scatterplots showed that codon usage in *P. gibbiceps* closely tracked the other taxa, with points clustering near the y = x line and very high correlations (0.993 ≤ r ≤ 1). In addition, the nearly identical codon totals and the concordant RSCU profiles indicated that mitochondrial codon-usage bias remained highly conserved in *P. gibbiceps* and related loricariids.

**Fig. 7:**
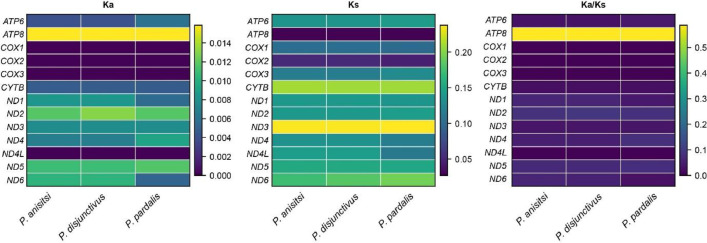
Heatmaps of Ka, Ks, and Ka/Ks values for the 13 mitochondrial protein-coding genes in *Pterygoplichthys gibbiceps* compared with other *Pterygoplichthys* species. The left, middle, and right panels show the nonsynonymous substitution rate (Ka), synonymous substitution rate (Ks), and Ka/Ks ratio, respectively, for the 13 mitochondrial protein-coding genes. Rows represent individual genes, and columns represent the compared species. Color intensity indicates the magnitude of each value, with lighter colors representing higher values.

Pairwise comparisons involving *P. gibbiceps* showed that, for most mitochondrial PCGs, synonymous substitution rates (Ks) were much higher than nonsynonymous substitution rates (Ka). In addition, all Ka/Ks values were below 1.0, indicating that these genes are mainly under purifying selection (Fig. 7). Among the 13 genes, *ATP8* showed the highest Ka and Ka/Ks values, highlighted in yellow on the heatmap, suggesting that it evolves faster than the other mitochondrial genes. However, its Ka/Ks value remained below 1.0, indicating no evidence of positive selection. In contrast, *COX1, COX2, COX3,* and *ND4L* had Ka/Ks values of zero, shown in dark purple, indicating very strong conservation at the amino acid level despite the presence of synonymous substitutions. The genes *ND3* and *CYTB* showed relatively high Ks values, indicated by green-to-yellow colors, suggesting greater synonymous divergence. Overall, the heatmap indicates that most mitochondrial genes in *P. gibbiceps* are under strong functional constraint, while *ATP8* appears to be the most variable gene among those analyzed.

Our mitogenome-based phylogeny recovered *Pterygoplichthys* as a strongly supported lineage within Hypostominae and placed *P. gibbiceps* inside the *Pterygoplichthys* clade, clustering most closely with *P. pardalis* and *P. disjunctivus.* This result was consistent with morphology-based systematics: Armbruster’s anatomical phylogeny showed that *Pterygoplichthys* and the historically used genera *Glyptoperichthys* and *Liposarcus* did not form separate monophyletic groups, and therefore classified *Glyptoperichthys* and *Liposarcus* as synonyms of *Pterygoplichthys* [14]. It also agreed with multilocus studies using mitochondrial and nuclear loci that reassessed genus-level relationships in Hypostominae and supported the placement of *Pterygoplichthys* within hypostomine lineages [15]. In addition, published mitogenomes of *P. pardalis* further showed a comparable mitochondrial gene framework for resolving relationships within Loricariidae, consistent with our genus-level clustering [7].

*Pterygoplichthys* species were morphologically similar and often closely related, which was sometimes insufficient for reliable identification; therefore, genetic data were increasingly used to resolve taxonomic uncertainty. By providing a mitogenome reference for *P. gibbiceps*, our data enabled the selection of highly variable mitochondrial regions to develop species-diagnostic markers that distinguished *P. gibbiceps* from congeners and traced maternal lineages in putative hybrids reported in *Pterygoplichthys* assemblages [16]. In practice, these markers were applied as (i) *COX1* DNA barcodes for routine identification and reference-library building, and (ii) targeted assays for rapid screening of environmental DNA in monitoring programs. Moreover, mitogenomes provided a large, linked marker set (13 PCGs, two rRNAs, 22 tRNAs, and a control region), yielded stronger phylogenetic signals than single mitochondrial genes, and improved relationship inference within *Pterygoplichthys* and other loricariids [11,12]. Although mitogenome-based studies were increasingly used in Loricariidae, mitogenomes for *Pterygoplichthys* still represented only a few species of the genus, so broader sampling was needed to stabilize phylogenies and better assess species limits and introgression.

In conclusion, the complete mitochondrial genome data of *P. gibbiceps* provide a valuable reference for comparative genomics and phylogenetic analyses within *Pterygoplichthys* and the family Loricariidae. As additional data become available, they will improve biogeographic reconstructions and enable more reliable inference of invasion sources and dispersal routes, thereby supporting monitoring and management of this invasion-prone armored catfish group.

## 5 Experimental Design, Materials and Methods

An adult specimen of *P. gibbiceps* (voucher number UMP_2025_10_VN) was sampled from Gia Lai Province, Vietnam, in October 2025. A small piece of muscle tissue was preserved in absolute ethanol and stored at −20 °C until DNA extraction. Total genomic DNA was extracted using the DNeasy Blood & Tissue Kit (69504, QIAGEN, USA) according to the manufacturer’s protocol. Library preparation for whole-genome sequencing was performed using the NEBNext Ultra II DNA Library Prep Kit for Illumina, and paired-end sequencing (150 bp) was conducted on an MGI BISEQ-50 platform at KTest Science Co. Ltd. (https://www.ktest.vn/, Ho Chi Minh City, Vietnam).

Adapter sequences and low-quality bases were removed with fastp v1.0.1 using default parameters and a minimum post-trimming read length of 100 bp [17]. The mitochondrial genome of *P. gibbiceps* was *de novo* assembled using NOVOPlasty v4.3.5 [18], with a seed sequence from the closely related loricariid mitogenome of *P. pardalis* (NC_060468) and key parameters including k-mer = 39, genome range = 14,000–20,000 bp, and insert size = 300 bp. Gene annotation was performed using MITOS2 on the Galaxy web server, which identified PCGs, tRNAs, rRNAs, and the putative control region. We manually inspected and refined the start and stop codons and gene boundaries using the BLAST tool (https://blast.ncbi.nlm.nih.gov/Blast.cgi) and by alignment with homologous mitogenomes of *P. pardalis* (NC_060468) and *P. anisitsi* (KT239003). In addition, tRNA genes were re-evaluated using tRNAscan-SE v2.0 (https://trna.ucsc.edu/tRNAscan-SE/). OGDRAW (https://chlorobox.mpimp-golm.mpg.de/OGDraw.html) was used to visualize the circular mitochondrial genome map.

Complete mitochondrial genomes of 29 loricariid taxa and one outgroup species from the family Callichthyidae were downloaded from the NCBI nucleotide database to assemble the phylogenetic dataset. Whole mitochondrial genome sequences were aligned using MAFFT v7.490, implemented in Geneious Prime v2024.0.5, and ambiguously aligned sites were removed using trimAl v1.5.0 (https://trimal.readthedocs.io/en/latest/). The best-fit nucleotide substitution model was selected using jModelTest v2.1.10 based on Akaike Information Criterion (AIC), which identified TVM+I+G as the optimal model (https://evomics.org/learning/phylogenetics/jmodeltest/). ML phylogeny was inferred in IQ-TREE (https://iqtree.github.io/) using the selected model with 1,000 ultrafast bootstrap replicates to evaluate node support. BI analysis was performed in MrBayes (https://ngphylogeny.fr/tools/tool/281/form) with 1,000,000 generations, sampling every 1,000 generations. The run was terminated when the average standard deviation of split frequencies fell below 0.01, and the first 20% of sampled trees were discarded as burn-in. Node supports were reported as bootstrap percentages for ML and posterior probabilities for BI. The resulting trees were visualized in Figtree v1.4.4 (https://tree.bio.ed.ac.uk/), with full annotation of support values and taxonomic classification.

Whole mitochondrial genomes were compared using progressiveMauve by aligning complete mitogenome sequences to identify locally collinear blocks (LCBs), evaluate genome synteny, and detect potential rearrangements among species. Relative synonymous codon usage values were calculated using CodonW (https://codonw.sourceforge.net/). Pairwise nonsynonymous (Ka) and synonymous (Ks) substitution rates for each mitochondrial protein-coding gene were estimated from codon-aligned nucleotide sequences using the Nei–Gojobori (NG86) method [19]. The Ka/Ks ratio was then calculated for each pairwise comparison to assess selective pressure acting on individual genes. The mitochondrial control region was extracted and analyzed for conserved motifs, repetitive elements, and secondary structure. Conserved regulatory motifs, including TAS-associated motifs and conserved sequence blocks, were identified by manual sequence inspection and comparison with previously reported mitochondrial control-region motifs. Simple sequence repeats were detected using BioPHP Microsatellite Repeat Finder (http://insilico.ehu.es/mini_tools/microsatellites/), whereas tandem repeats were identified using Tandem Repeats Finder v4.09.1 (https://tandem.bu.edu/trf/trf.html). The secondary structure of the control region was predicted using the RNAfold web server (http://rna.tbi.univie.ac.at//cgi-bin/RNAWebSuite/RNAfold.cgi).

## Limitations

• Single-individual sampling: The mitogenome was assembled from a single individual of *P. gibbiceps* collected at one locality. Consequently, the dataset does not capture intraspecific mitochondrial variation across the species' broader geographic range.
• Short-read sequencing only: The mitochondrial genome was exclusively generated from short-read Illumina data. The lack of long-read sequencing data may limit the independent assessment of structural features and repetitive regions.
• Mitochondrial genome only: The dataset exclusively focuses on the mitochondrial genome. Nuclear genomic and transcriptomic data were not provided, restricting the dataset to maternal lineage markers.
• Limitation of compared data: The limited availability of *Pterygoplichthys* mitochondrial genomes restricted our analysis to only a few species within this genus.

## Ethics Statement

All sampling procedures and experimental protocols involving *P. gibbiceps* complied with institutional, national, and international guidelines for the care and use of animals. One wild adult specimen (voucher ID: UMP_2025_10_VN, sex: male) was collected in October 2025 from Dak Lak Province. The species is not listed as a protected or endangered taxon under the current wildlife regulations.

## CRediT Author Statement

**Thu Thao Thi Huynh:** Conceptualization, methodology, resources, data curation, and writing of the original draft; **Phi Anh Nguyen Ngoc:** Conceptualization, methodology, investigation, and validation; **Nga Thi Nguyen:** Conceptualization, methodology, resources, data curation, validation, writing, review, and editing; **Minh Trong Quang:** Conceptualization, methodology, validation, visualization, review, and editing.

## Data Availability

Genbank, NCBIPX583372 (Reference data).

## Appendix Supplementary materials

Image, application 1
