# Supplementary material for: Complete mitochondrial genome sequence data of Pterygoplichthys gibbiceps (Actinopterygii, Loricariidae)
Source: Data Brief. 2026 Jun 11;67:112963. doi: 10.1016/j.dib.2026.112963 (PMC13285683; doi:10.1016/j.dib.2026.112963)
Supplement: Supplementary file 1 [file mmc1.docx]

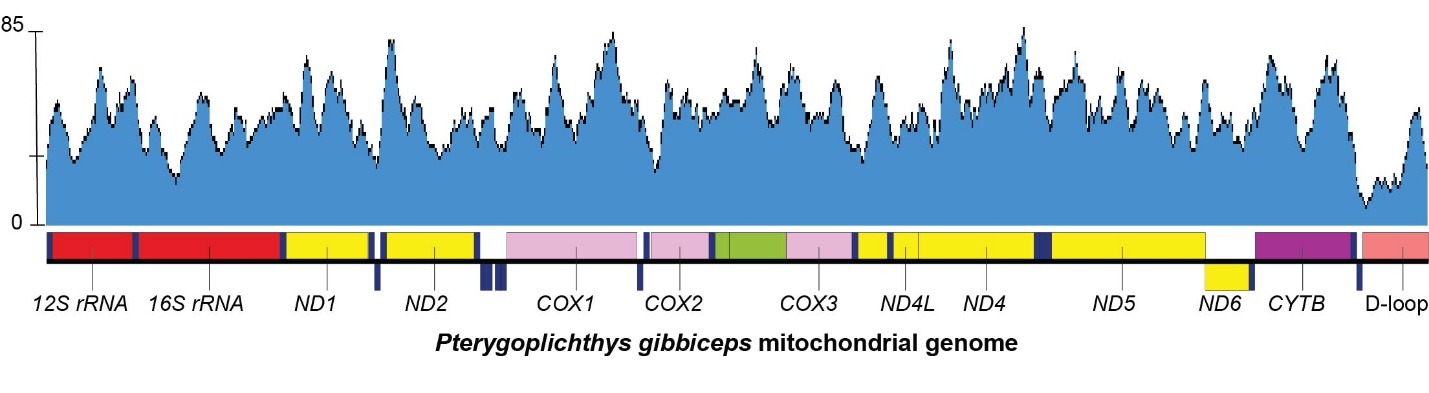
**Fig. S1.** Sequencing coverage across the mitochondrial genome of *Pterygoplichthys gibbiceps* (minimal: 22×; maximal: 85×; and average: 54×). The blue shaded area represents the read depth across the genome. The x-axis shows genomic organization, and the y-axis indicates sequencing depth. Annotated mitochondrial genes are displayed below the coverage plot, including ribosomal RNA genes, protein-coding genes, transfer RNA genes, and D-loop.


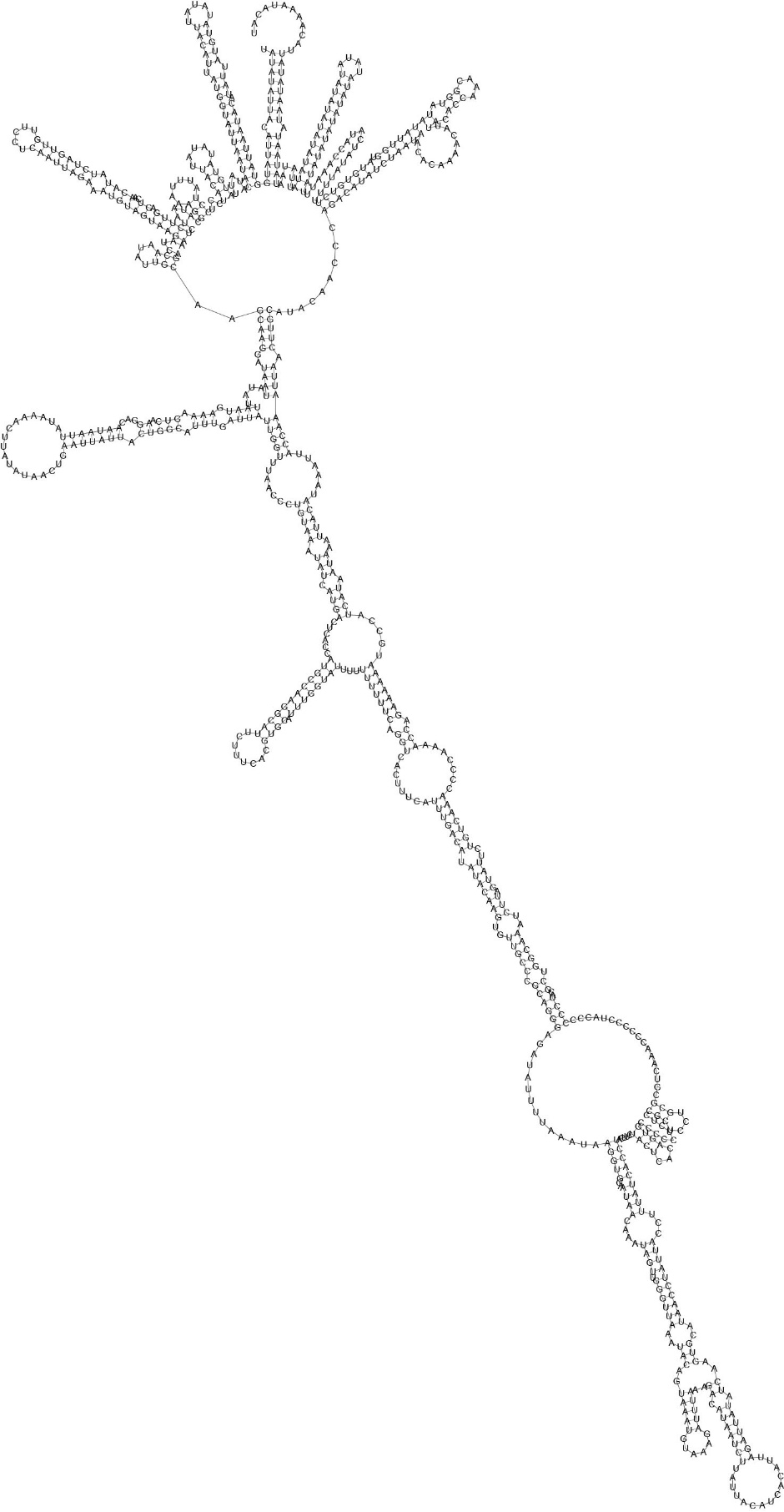


**Fig. S2.** Predicted secondary structure of the mitochondrial control region of *Pterygoplichthys gibbiceps*. The secondary structure of the 785-bp mitochondrial control region was predicted and visualized using the RNAfold Web Server.
